# High-throughput analysis of *ANRIL* circRNA isoforms in human pancreatic islets

**DOI:** 10.1038/s41598-022-11668-w

**Published:** 2022-05-11

**Authors:** Hannah J. MacMillan, Yahui Kong, Ezequiel Calvo-Roitberg, Laura C. Alonso, Athma A. Pai

**Affiliations:** 1 RNA Therapeutics Institute, University of Massachusetts Chan Medical School, Worcester, MA 01605 USA; 2 UMass Diabetes Center of Excellence, University of Massachusetts Chan Medical School, Worcester, MA 01605 USA; 3 Division of Endocrinology, Diabetes and Metabolism, Weill Cornell Medicine, New York, NY 10021 USA; 4 Weill Center for Metabolic Health, Weill Cornell Medicine, New York, NY 10021 USA; 5 Present Address: Curia Global, Inc., Hopkinton, MA 01748 USA

**Keywords:** Non-coding RNAs, Type 2 diabetes, Genetics

## Abstract

The antisense non-coding RNA in the INK locus (*ANRIL*) is a hotspot for genetic variants associated with cardiometabolic disease. We recently found increased *ANRIL* abundance in human pancreatic islets from donors with certain Type II Diabetes (T2D) risk-SNPs, including a T2D risk-SNP located within *ANRIL* exon 2 associated with beta cell proliferation. Recent studies have found that expression of circular species of *ANRIL* is linked to the regulation of cardiovascular phenotypes. Less is known about how the abundance of circular *ANRIL* may influence T2D phenotypes. Herein, we sequence circular RNA in pancreatic islets to characterize circular isoforms of *ANRIL*. We identify several consistently expressed circular *ANRIL* isoforms whose expression is correlated across dozens of individuals and characterize *ANRIL* splice sites that are commonly involved in back-splicing. We find that samples with the T2D risk allele in *ANRIL* exon 2 had higher ratios of circular to linear *ANRIL* compared to protective-allele carriers, and that higher circular:linear *ANRIL* was associated with decreased beta cell proliferation. Our study points to a combined involvement of both linear and circular *ANRIL* species in T2D phenotypes and opens the door for future studies of the molecular mechanisms by which *ANRIL* impacts cellular function in pancreatic islets.

## Introduction

Type 2 diabetes (T2D) is a worldwide problem of increasing social and economic significance. Although obesity and insulin resistance are important contributors, an essential common element required for T2D progression is failure of pancreatic islets to produce sufficient circulating insulin to meet demand^1^. Large scale efforts have identified > 400 genomic regions that are associated with T2D risk in human populations^2^; however, identifying the underlying molecular mechanisms has been slower than hoped.

Polymorphisms at the *CDKN2A/B* gene locus at chromosome 9p21 are prominently associated with diabetes risk in disparate human populations and across a range of T2D-related syndromes^3^. Although the gene products at *CDKN2A/B* play roles in many cell types, strong evidence links *CDKN2A/B* T2D risk-polymorphisms specifically to impaired insulin secretory function in humans, pointing to impact on pancreatic islets as the causative mechanism by which this gene locus drives diabetes risk^4–10^.

The *CDKN2A/B* locus contains two protein-coding genes: *CDKN2A*, which encodes p14^ARF^ and p16^INK4A^, and *CDKN2B* which encodes p15^INK4B^, and a long noncoding RNA called *CDKN2B-AS* (antisense to CDKN2B) or *ANRIL*^11^. Although p14^ARF^, p16^INK4A^ and p15^INK4B^ play multiple biological roles in mouse and human β-cells^11^ and were widely assumed to mediate the T2D risk transmitted by risk-SNPs at this locus, a study we performed analyzing *CDKN2A/B* risk-SNP impact on human islets showed a stronger association with *ANRIL* than with the protein-coding genes^12^. Specifically, in 95 unique nondiabetic human islet samples, two T2D risk alleles at *CDKN2A/B* were differentially associated with *ANRIL* abundance in an age-dependent manner^12^. Intriguingly, carriers of a T2D risk-SNP located within an exon of *ANRIL* had a reduced β-cell proliferation index than carriers of the protective allele^12^. These findings outline an urgent need to better understand the biology of *ANRIL* in pancreatic islets.

*ANRIL* is a low-abundance but widely expressed lncRNA that is implicated in multiple disease states and diverse cell types^13–15^. Containing at least 22 exons^16^, many *ANRIL* isoforms have been described, including circular forms^13,17–21^, and are distributed across both nuclear and cytoplasmic compartments^13,18,22–27^. *ANRIL* expression has previously been associated with cell growth, proliferation, and apoptosis phenotypes in cancer, senescence and cardiovascular disease cellular models^14,28,29^. Intriguingly, increasing evidence suggests that there may be cross-talk or divergent functionality between linear and circular isoforms of *ANRIL*. Linear *ANRIL* (lin*ANRIL*) is thought to bind to PRC1/2 and act as a molecular scaffold guiding epigenetic complexes to promote cell adhesion, proliferation, and apoptosis^21^. However, circular *ANRIL* (circ*ANRIL*) instead appears to compete with ribosomal RNA (rRNA) for binding with PES1—a component of the PeBoW complex that plays a role in cell proliferation via pre-rRNA processing—resulting in impaired rRNA maturation and inhibition of proliferation^20,21^. Consequently, the relative abundance of lin*ANRIL* and circ*ANRIL* may regulate proliferative or apoptotic phenotypes associated with metabolic disease and has been implicated as such in models of atherosclerosis risk^17,20^, cancer proliferation^18^, and epithelial cell response to stimulus or injury^30^.

Multiple other antisense long non-coding RNAs (lncRNAs) have been shown to play a pivotal role in the regulation of gene expression and disease etiology, often by serving as scaffolds for the recruitment or occlusion of gene regulatory machinery^31^. For instance, linear antisense lncRNAs *HOTAIR*^32^ and *APOA1-AS*^33^ may contribute to the progression of cancer and cardiovascular disease, respectively, through the dysregulation of histone modification patterns. Reminiscent of lin*ANRIL*, *HOTAIR* aids in the recruitment of the PRC2 complex to epigenetically silence cancer suppressor genes^32^, while *APOA1-AS* recruits a wider suite of multiple histone modifying enzymes to alter gene expression programs^33^. Similarly, the tumor suppressor mRNA gene *SCRIB* also produces multiple antisense circRNAs including *circSCRIB*, which has been implicated in cancer progression through the suppression of splicing and translation of its parent gene mRNA product^34^.

Despite the increasing recognition that circular forms of *ANRIL* or other lncRNAs may play a role in disease biology, little is known about the mechanisms that underlie circ*ANRIL* expression. Circular RNAs are produced by non-canonical splicing between a downstream 5’ splice site with an upstream 3’ splice site, in a mechanism called back-splicing. Since these molecules have no 5’ cap or 3’ polyA tail, they are not believed to be translated and cannot be targeted by exonucleolytic degradation. Thus, circ*ANRIL* isoforms are thought to have greater stability than lin*ANRIL* species^17,20,30^ and, consistently, have been seen at greater levels than lin*ANRIL* in immune cells^17,20^. The handful of studies that have characterized *ANRIL* exons involved in back-splicing events have not identified any cis-elements that may promote back-splicing. However, most of these studies lacked the ability to perform a fully unbiased identification of circ*ANRIL* isoforms and their relative expression levels, potentially obscuring the full picture of exons and genomic elements that may contribute to *ANRIL* circularization. Here, we use high-throughput sequencing to identify and quantify circular *ANRIL* isoforms in human pancreatic islets.

## Results

Previous studies in immune, cancer, and epithelial cells have identified a wide range of circ*ANRIL* isoforms that are expressed in different conditions^17,18,20,30^. However, such an analysis has not been conducted in pancreatic islets, despite evidence that circular and linear *ANRIL* expression may be implicated in diabetes phenotypes. The aim of our study is to systematically characterize circ*ANRIL* isoforms and their regulation in non-diabetic human islet tissue, with the goal of defining the circ*ANRIL* landscape in this critical metabolic tissue.

### Identifying circular isoforms of *ANRIL* in pancreatic islets

We first set out to identify pancreatic islet circ*ANRIL* isoforms in an unbiased fashion. To do so, we obtained five frozen islet preparations from the Integrated Islet Distribution Program (Methods; Table S1) and conducted RNA-seq after digestion with RNase R (which enriches for circRNA by degrading linear RNA molecules), paired with mock digested samples to obtain total RNA abundance for each sample. To identify back-spliced exon junctions indicative of circularization, we used the circRNA analysis package Circexplorer2^35^ after linear read alignment with STAR^36^. Supporting efficacy of this approach, the RNase R treated samples had over 4.5 × more back-spliced junctions (BSJ) reads than the untreated samples (0.65% vs 0.14% of all reads, respectively; Fig. S1A). RNA-seq data across different islet samples were highly consistent, with an average gene expression correlation greater than 0.95 within RNase R treated samples and 0.94 within untreated samples (Pearson correlations, Fig. S1B). Our data correlated well with publicly available pooled human islet RNA-seq from Haque et al.^37^, providing additional confidence to our sample preparation. Thus, we decided to include the Haque et. al. data in our downstream analyses to provide more statistical power for circ*ANRIL* isoform identification. To identify circ*ANRIL* isoforms independent of known linear isoforms, which contain different subsets and combinations of exons, we collapsed all annotated linear exons into a meta-isoform and re-numbered exons based on this meta-isoform for reference in all downstream analyses (Fig. S1C).

**Figure 1:**
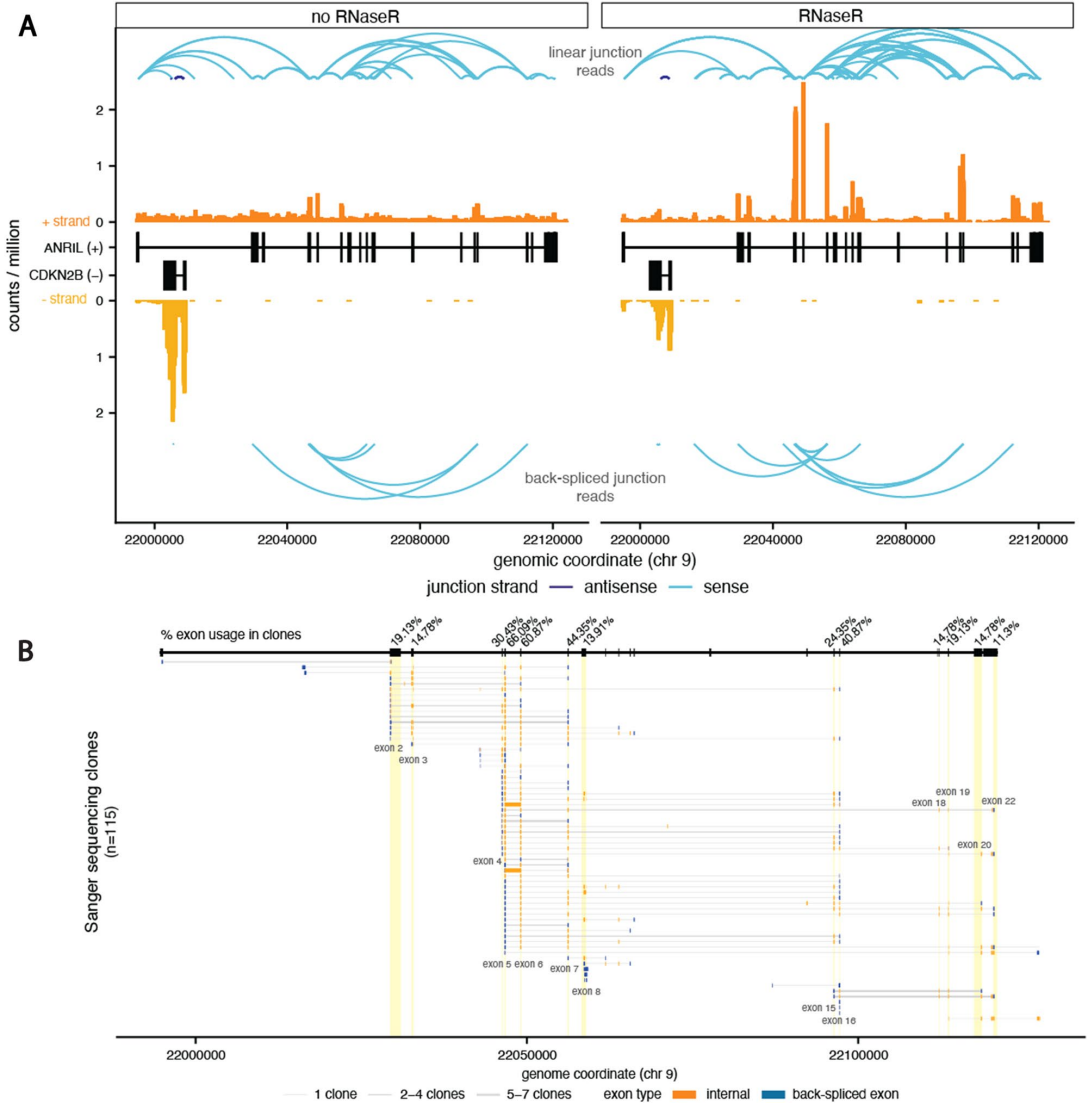
Transcription of *ANRIL* locus results in diverse circular RNAs. (**A**) RNA-seq data from the *CDKN2B/CDKN2B-AS* locus from control (*left*) and RNaseR treated (*right*) RNA. Exonic read coverage is in orange shades and splice junction reads are in blue, differentiated by linear (*top*, from STAR alignment) and back-spliced (*bottom*, from CIRCexplorer2 alignment) junction reads. All read counts are normalized by the total number of reads in the libraries (**B**) Visualization of individual fragments identified by Sanger sequencing, with each row representing one isoform and the thickness of the lines representing the number of corresponding sequencing clones. Exons involved in back-splicing are represented in blue.

Upon RNase R treatment, we observed an enrichment of circRNA at the *ANRIL* locus (Fig. 1A). Though there were almost twice as many linear junction reads in the untreated samples, there was a 2.65 fold increase in BSJs at the *ANRIL* locus in the RNase R treated samples (Table S2). Interestingly, there were also more linear junction pairs of splice sites in the RNase R samples, perhaps indicating greater alternative splicing in circRNA molecules. Reassuringly, read coverage for the antisense *CDKN2B* mRNA was lower in the RNase R treated samples and no BSJ reads mapped to this locus. A number of *ANRIL* exons had greater read coverage in the RNase R samples, suggesting that *ANRIL* circularization might preferentially involve specific *ANRIL* exons. To quantify circRNA abundance we used the CIRIquant package^38^, which performs a direct comparison between linear and circular RNA species and accounts for both RNase R and untreated samples to calculate an adjusted BSJ count per locus (Table S3). After removing BSJs that occurred in only one individual or were only supported by one read, we identified 17 high confidence circ*ANRIL* BSJs that used splice sites corresponding to 11 different exons and three intragenic regions (Fig. S1D). To validate our high-throughput sequencing data, we designed divergent primers in 8 *ANRIL* exons and conducted PCR, cloning, and Sanger sequencing for 115 clones in endoC-βH1 cells (Fig. 1B). The immortalized endoC-βH1 cell line is an established human beta cell line^39^ that is complementary to primary islets, which are composed of mixed α and β cells, and is a renewable cell population that enables in-depth molecular and cellular characterizations. While the lower throughput sequencing identified 35 back-splicing events involving a greater number of exons, 59% of our RNA-seq-based BSJs were validated, including all of the BSJs with the greatest read coverage (Fig. S1E). Interestingly, Sanger sequencing clones also identified extensive *ANRIL* cryptic splice site and exon usage, with a number of intronic sites involved in back-splicing and intronic sequences spliced into circ*ANRIL* isoforms (Table S4).

### Consistent expression of specific circ*ANRIL* isoforms

**Figure 2:**
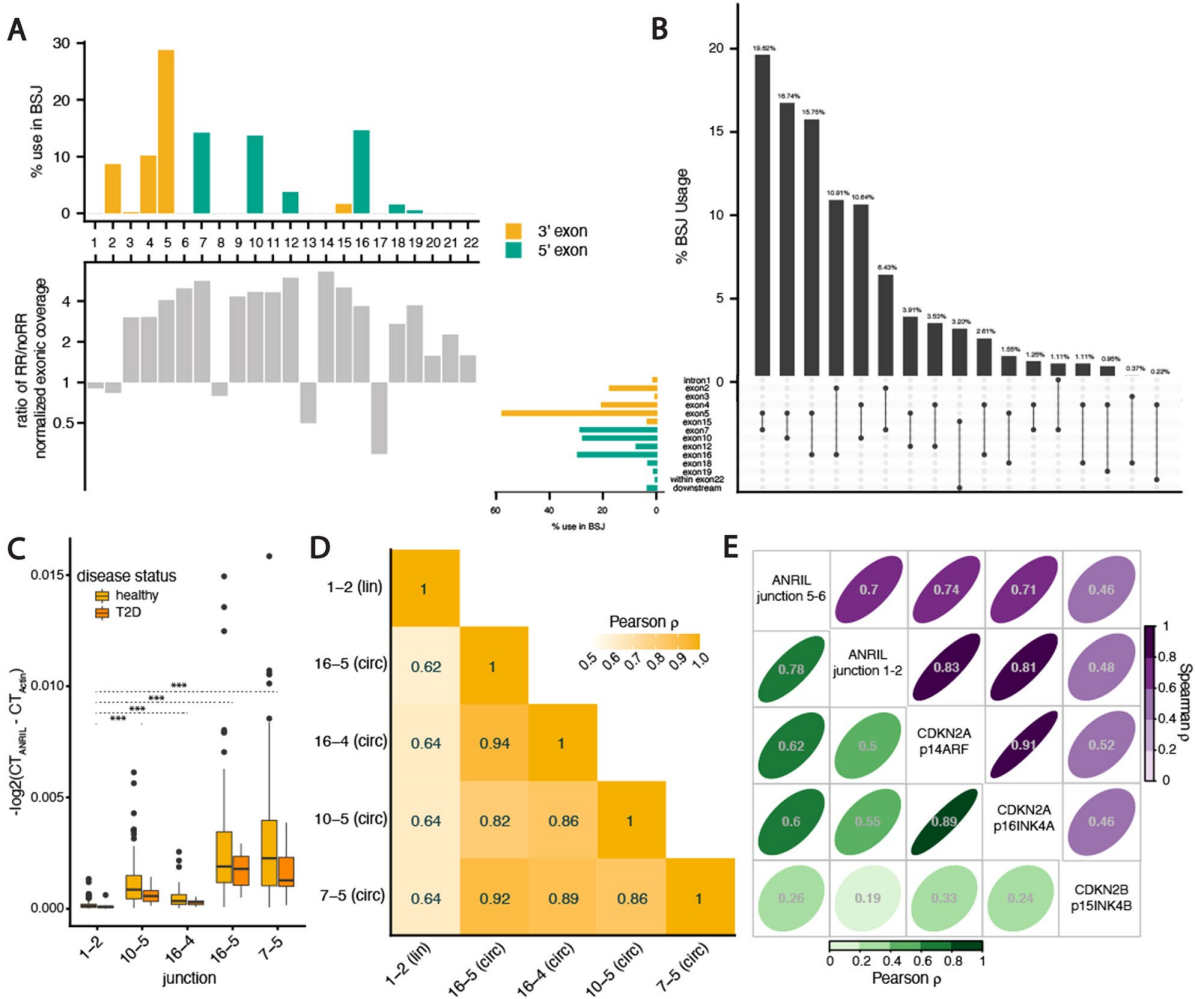
Consistent expression of specific circ*ANRIL* isoforms. (**A**) Exon-specific involvement in back-spliced junctions (*top*) and fold-change in expression between RNaseR treated and control samples (*bottom*, normalized for library depth). (**B**) Frequency of splice site pairing in back-spliced junctions, with proportional usage (*top*) for each BSJ (*bottom*), with proportional usage of individual exons on the left. (**C**) Pairwise Pearson correlations between linear (junction 1–2) and circular *ANRIL* expression across 122 islet samples. (**D**) RT-PCR quantification of linear and circular isoforms across 122 islet preparations. (**E**) Pairwise Pearson correlations (*bottom left*) and Spearman rank correlations (*top right*) between linear *ANRIL* (junction 1–2), circular *ANRIL* (junction 5–6), *CDKN2A* isoforms, and *CDKN2B* abundance across 122 islet preparations.

Next, we capitalized on the long-read Sanger sequencing data to gain insight into the internal exon structure of circ*ANRIL* isoforms. Since Sanger sequencing data is biased by the number of clones sequenced from each primer set (which are placed in exons that are likely not equally represented across circ*ANRIL* isoforms), these data do not provide quantitative information about isoform expression. Nevertheless, we observed a striking concordance: exons that were present in the majority of Sanger clones were also overrepresented in the RNase R RNA-seq data, measured by the fold change in exonic coverage between RNase R and untreated samples (Fig. 2A) These overrepresented exons also tended to fall within the linear boundaries of exons that were most frequently used in back-splicing reactions, including exons 2, 4 and 5 as 3’ acceptors and exons 7, 10, and 16 as 5’ donors in the back-splicing reactions.

The overrepresentation of specific *ANRIL* exons in BSJs and internal circRNA structure suggests preferential expression of particular circ*ANRIL* isoforms. Indeed, we found that several BSJs occurred at higher frequency (Fig. 2B), with the exon7-exon5 pairing alone contributing almost 20% of BSJ reads, 4 other pairings contributing more than 10% of BSJ reads, and exon5 acting as a 3’ acceptor site in more than half of the BSJ reads. Notably, three isoforms previously seen to be predominantly expressed across immune and cancer cells types^17,18,20^, 7–5, 10–5 and 16–5, were the three highest expressed circ*ANRIL* isoforms in islets. To validate relative expression of the most predominant circ*ANRIL* isoforms, we used qRT-PCR to quantify the enrichment of 4 BSJ junctions (7–5, 10–5, 16–4, and 16–5), 2 linear junctions (1–2 and 5–6) and two predominantly linear housekeeping genes following RNase R treatment in endoC-βH1 cells. The relative abundance of BSJ-containing RNA remained high after RNase R treatment, while the housekeeping genes and linear 1–2 junction were expressed at significantly lower levels **(**all Mann–Whitney U test *p*-values < 0.001, Fig. S2A). Interestingly, relative expression of the linear 5–6 junction was similarly abundant to the BSJ containing RNA, indicating that exons 5 and 6 together may be primarily present in circRNA and serve as a good proxy for circRNA expression. This is consistent with the notable increase in reads for exons 5 and 6 after RNase R treatment (Fig. 1A) and presence of these exons in greater than 65% of Sanger sequenced clones (Fig. 1B).

The skewed frequency of splice site pairing suggested that *ANRIL* back-splicing may be a regulated event. Since our RNA-seq data were not powered to evaluate individual-specific expression levels across isoforms, we turned to a larger cohort of 95 islet preparations in which we had previously characterized lin*ANRIL* expression^12^ and added 27 samples for a total of 122 samples (Methods; Table S5). With this larger set of samples, we used qRT-PCR to measure expression of linear *ANRIL*, using an assay for exon 1–2 found predominantly in lin*ANRIL*, and specific circ*ANRIL* isoforms identified with the RNA-seq data (Methods). First, we confirmed that circ*ANRIL* isoforms are expressed at significantly higher levels than linear *ANRIL* on average across these islet samples (all T-test *p*-values < 10^–8^; Fig. 2C)^17,20,40^. We saw no significant differences between *ANRIL* isoform abundances between the non-diabetic and 10 diabetic samples in our dataset, however our power to detect small to moderate differences was likely limited by the small number of diabetic samples we were able to obtain. Thus, for all further analyses, we used the combined set of non-diabetic and diabetic individuals. We found that circ*ANRIL* isoforms correlated better with other circ*ANRIL* isoforms (all Pearson R > 0.8) than with linear *ANRIL* (average Pearson R = 0.64; Fig. 2D), suggesting independent regulation of back-splicing across individuals after transcription of *ANRIL*. To understand the regulation of circ*ANRIL*, we examined the Spearman rank correlations between circ*ANRIL* expression (measured as junctions per million, JPM; Methods), and the expression of other RNAs transcribed at the locus. Across the 5 islet samples on which we performed RNA-seq, we found a lower correlation between linear and circ*ANRIL* than with other genes transcribed at the locus (Fig. S2B), including the antisense mRNA *CDKN2B* and the upstream divergently transcribed mRNA *CDKN2A*. To gain more power, we used qRT-PCR measurements in the larger cohort of 112 islet samples and again found that linear and circ*ANRIL* had a lower rank correlation with each other than other genes transcribed at the locus (Spearman R = 0.7; Fig. 2E). Consistent with our previous results^12^, the expression of both linear and circular *ANRIL* were more highly correlated to the expression of *CDKN2A* isoforms than *CDKN2B*, suggesting that *ANRIL* and *CDKN2A* transcription might be co-regulated in islets by the same promoter or enhancer elements. Notably, while the Spearman rank correlations are high, the lower Pearson correlations suggest differences in transcriptional output from the *ANRIL* and *CDKN2A* loci and again point to the regulation of circ*ANRIL* expression across individuals. Intriguingly, both protein isoforms encoded by *CDKN2A*, ARF (p14) and INK4A (p16), function as tumor suppressors by regulating cell proliferation, linking CDKN2A protein function to the cellular phenotype observed to be regulated by both linear and circular ANRIL^21^. Finally, the low correlation between *ANRIL* and *CDKN2B* expression levels does not support a model in which *ANRIL* RNA directly regulates *CDKN2B* levels through complementary binding interactions.

### Local sequence elements may mediate circ*ANRIL* production

**Figure 3:**
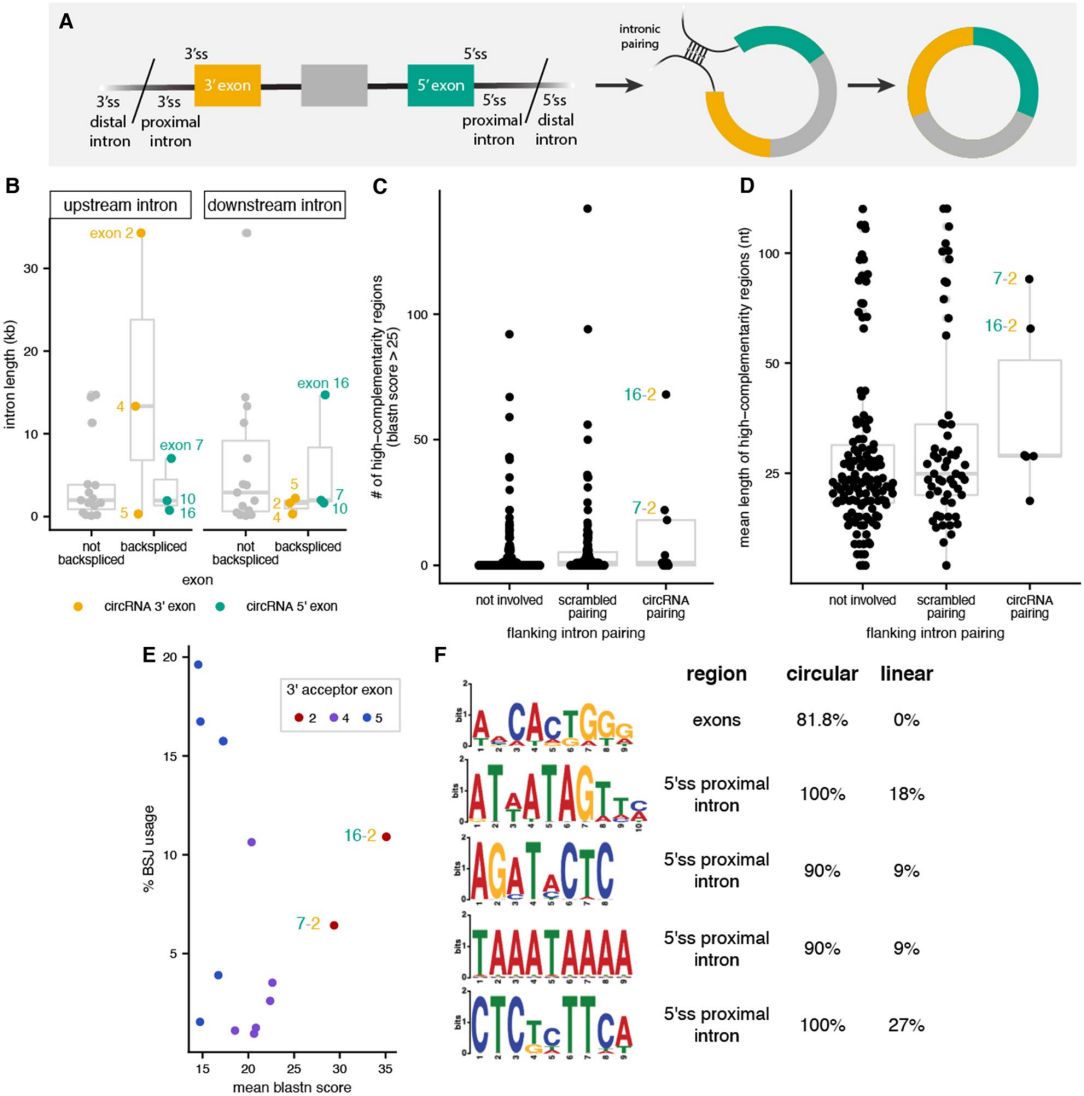
*ANRIL* back-splicing is associated with sequence features. (**A**) Schematic for BSJ involved 3’ and 5’ splice site (ss) proximal introns used as input for motif enrichment (*left*) and intron pairing (*right*). (**B**) Distribution of intron lengths (*y-axis*) for 3’ and 5’ back-spliced exons (*yellow* and *green*, respectively) and exons not involved in back-splicing (*grey*). (**C**) Distribution of the number of high complementarity (blastn > 25) regions (*y-axis*) within pairs of flanking introns. (**D**) Distribution of length for high complementarity regions (*y-axis*) within pairs of flanking introns. (**E**) Correlation between mean complementarity scores from blastn for high complementarity regions (*x-axis*) and %BSJ isoform usage (*y-axis*) for frequently used 3’ exons. (**F**) Sequence motifs enriched in *ANRIL* BSJ-involved exons or flanking introns, with percent occurrence in circular or non-circular (linear) regions indicated on the right.

We observed that back-splicing is more likely to occur at a small number of splice sites at the *ANRIL* locus. Furthermore, the expression levels of these circular isoforms were highly correlated with each other but not with the levels of linear *ANRIL*. Taken together, these results suggest co- or post-transcriptional regulation of back-splicing at the *ANRIL* locus. To investigate potential cis-elements that regulate the formation of circRNAs, we focused on 5 features: (1) splice site strength, (2) intron length, (3) repeat regions, (4) sequence complementarity and (5) enriched motifs. For these analyses, we differentiated between circ*ANRIL* 3’ exons, which are associated with the 3’ acceptor participating in the back-splicing reaction, and 5’ exons, which are associated with the back-spliced 5’ donor (Fig. 3A). First, we observed that 5’ splice sites of circ*ANRIL* 3’ exons were significantly weaker than those of non-BSJ and 5’ exons (bootstrapped *p*-value = 0.03, Methods, Fig. S3A), suggesting that 3’ exons involved in BSJs may not be well defined by canonical exon-definition pathways and thus be more likely to have unpaired 3’ splice sites available for back-splicing with downstream exons^41^.

Previous studies have seen that back-spliced exons tend to be flanked by longer introns^42,43^. Consistently, the introns upstream of 3’ exons 2 and 4 and downstream of exon 16 are among the longest annotated *ANRIL* introns (Fig. 3B) and could potentially harbor more sequence elements that promote or regulate *ANRIL* back-splicing events. One feature proposed to promote back-splicing is complementarity-mediated pairing between introns flanking back-spliced exons^42,44,45^, which brings together the upstream 3’ splice site and downstream 5’ splice site in 3D space (Fig. 3A). Contrary to what has been observed for other circRNAs^46–48^, we did not observe enrichment of known repeat regions within *ANRIL* introns flanking sites involved in back-splicing reactions (Fig. S3B). Thus, we instead looked for non-repeat complementary regions in introns using blastn (Methods). We observe that the pairing between introns flanking exons 7–2 and 16–2 have among the most high-scoring complementary regions (Fig. 3C) and the longest average length of complementary regions (Fig. 3D) of any pairwise combinations of *ANRIL* introns. This suggests that the use of exon 2 as a 3’ exon in a back-splicing event may be mediated purely by sequence complementarity, consistent with the idea that exon 2 appears to mostly appear in linear *ANRIL* and appears in less than 10% of BSJs. Indeed, we observed that the higher-scoring 16–2 pairing was used more in BSJs than the lower-scoring 7–2 pairing (Fig. 3E), a correlation that was not observed for the intronic pairings of other circ*ANRIL* 3’ exons.

Lastly, back-splicing may be directly regulated by splicing factors that bind to BSJ-involved exons or flanking introns^49^, so we looked for enrichment of sequence motifs within BSJ involved regions relative to non-involved regions (Methods). We identified 5 motifs that were present in more than 80% of BSJ-involved exons or introns—one in exons and the other four in the intronic region proximal to BSJ-involved 5’ splice sites (Figs. 3F, S3C). Interestingly, the motif enriched in BSJ-involved exons matches the known motif for the SFRS13A (aka SRSF10) SR-protein splicing factor, which has previously been shown to promote back-splicing in humans, drosophila, and murine models^50–52^.

### circ*ANRIL* exons have more miRNA target sites

**Figure 4:**
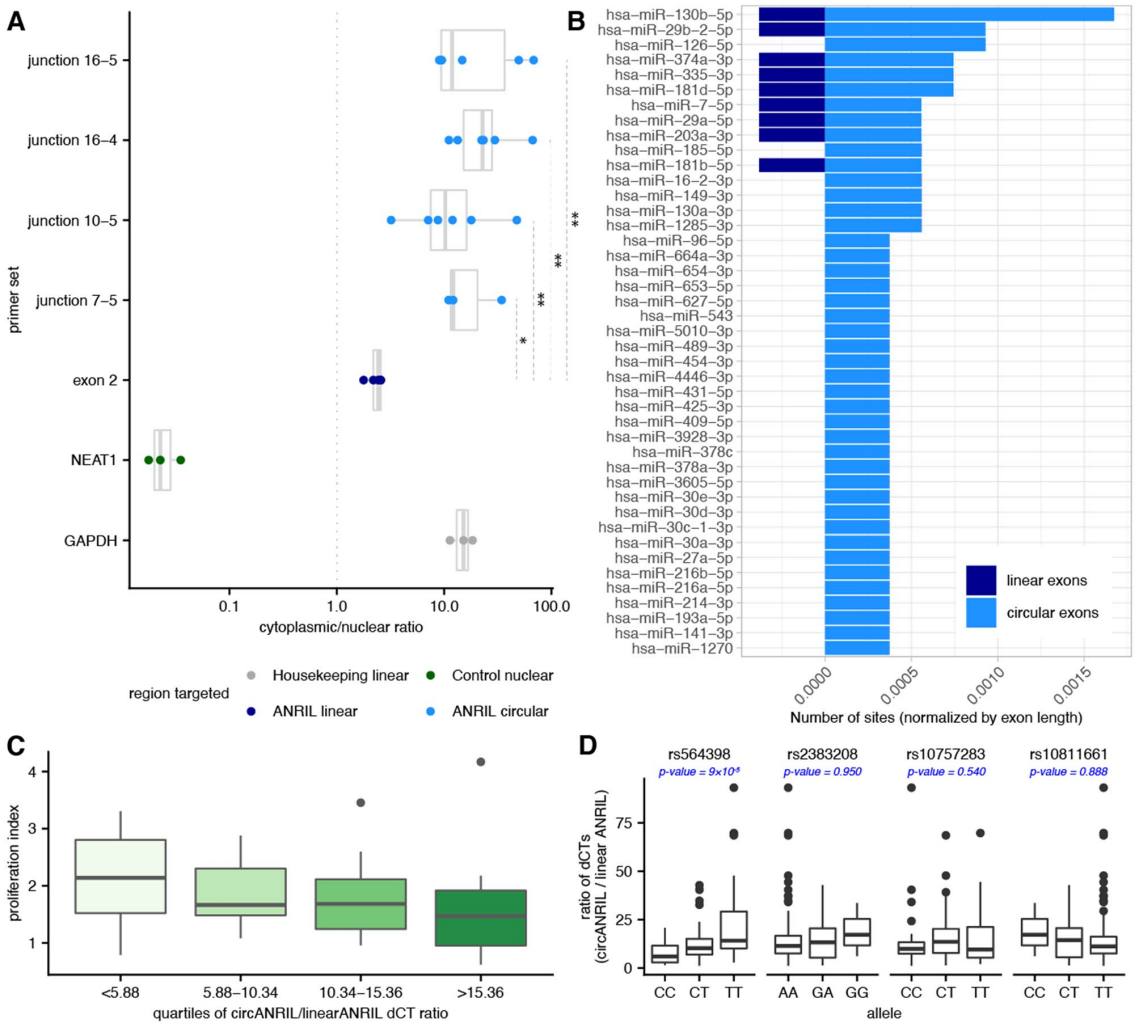
circ*ANRIL* is associated with cytoplasmic functions. (**A**) Ratio of cytoplasmic to nuclear abundance (*x-axis*) as measured by RT-PCR quantification with primer sets targeting a housekeeping gene (*grey*), a nuclear control gene (*green*), lin*ANRIL* (*light blue*) and circ*ANRIL* junctions (*dark blue*). (**B**) Number of miRNA target sites (normalized by exon length, *x-axis*) in *ANRIL* circular exons (*light blue*) vs *ANRIL* linear exons (*dark blue*) for miRNAs with more sites in circ*ANRIL* exons. (**C**) Distribution of proliferation indices (*y-axis*) for quartiles of circ*ANRIL*/lin*ANRIL* ratios (*x-axis*) as measured by RT-PCR across 45 islet preparations. (**D**) Association between genotypes and circ*ANRIL*/lin*ANRIL* ratios for 4 disease-associated SNPs at the *ANRIL* locus across 122 islet preparations. P-values are computed from a linear regression and Benjamini–Hochberg corrected.

Our results support regulated expression of *ANRIL* circRNA in human pancreatic islets. Thus, with our cell-type specific catalog of *ANRIL* isoforms, we next aimed to investigate the potential functional roles for circ*ANRIL* in islet cells. We first asked whether *ANRIL* RNA was differentially localized across cellular compartments using endoC-βH1 cells, in which subcellular fractionation experiments are more feasible than primary islets. Consistent with previous observations^17,20^, we found that circ*ANRIL* isoforms were predominantly cytoplasmic, with tenfold greater abundance in the cytoplasm on average than in the nucleus (Fig. 4A). Cytoplasmic to nuclear ratios of circ*ANRIL* were also significantly higher than linear *ANRIL* (all Mann–Whitney U *p*-values < 0.05), which was approximately equally abundant in the nucleus and cytoplasm.

circRNAs that are abundant in the cytoplasm have been previously implicated as cellular sponges acting to sequester RNA binding proteins (RBP) or miRNAs away from other regulatory targets^49,53,54^. Since we had not found any cytoplasm RBP motifs in circ*ANRIL* exons, we turned our attention to miRNA target sites. We looked for the presence of miRNA target sites in *ANRIL* exons frequently used in circ*ANRIL* isoforms (both participating in BSJs or internal exons) relative to exons more frequently seen in linear *ANRIL*, conditioning on miRNAs that are expressed in islet cells (mean RPM > 5 in the miRmine database; Methods). We find 43 miRNAs with more target sites in circ*ANRIL* exons than in lin*ANRIL* exons, while only 18 have more target sites in lin*ANRIL* exons (normalized by respective total exon lengths, Figs. 4B, S4A; Table S6). It is important to note, however, that the total length of exons present in circ*ANRIL* is approximately double the length of exons present exclusively in lin*ANRIL*, providing greater chances for miRNA target sites to occur purely by chance. Previous experimental investigations have identified *ANRIL* RNA association with miR-7-5p (in periodontal ligament stem cells^55^), miR-9 (kidney epithelial cells^56^), miR-622 (brain microvascular endothelial cells^57^), miR-140-5p (lung epithelial cells^58^). miR-622 was not expressed in islets and of the remaining 3 miRNAs, only miR-7-5p was found to have more target sites in circ*ANRIL* exons. The miRNA with the highest number of sites in *ANRIL* exons, miR-130b-5p, has 4.38 fold more sites in circular exons than linear exons. miR-130b-5p is known to target and downregulate several oncogenes and was seen to be downregulated itself in pancreatic cancers^59^, in which *ANRIL* is overexpressed^60^.

### Ratio of circular/linear *ANRIL* is associated with beta cell proliferation and diabetes risk genotype

The balance between linear and circular *ANRIL* expression has previously been associated with cell proliferation and apoptosis phenotypes in cancer and atherosclerosis cell models^17,18,20,61,62^. Thus, we wanted to understand whether the consistently expressed, cytoplasmic circ*ANRIL* isoforms in islet cells may play a role in islet biology or diabetes-relevant cellular phenotypes. We again turned to the expanded cohort of previously published primary human islet preparations to investigate two important aspects of islet function to *ANRIL* expression: insulin secretion and β-cell proliferation^12^. We did not find a clear relationship between insulin secretion—measured by a glucose stimulation index (Methods)—and levels of lin*ANRIL*, circ*ANRIL*, or the relative ratio of circular to linear *ANRIL* abundance (circ/lin*ANRIL*; Figs. S4B). However, we observed a clear monotonic relationship between the ratio of circ/lin*ANRIL* and the β-cell proliferation index (described previously in Kong et al. 2018^12^; Methods). Insulin-positive cells in islet preparations expressing the lowest circ/lin*ANRIL* ratio had a significantly higher proliferation index than preparations with higher circ/lin*ANRIL* (Fig. 4C; Table S7), but no relationship was observed between proliferation and abundance of lin*ANRIL* or circ*ANRIL* isoforms alone. Previous studies have shown that over-expressing circ*ANRIL* isoforms decreased proliferation in other cell types^20,40,57^, suggesting that the relationship between circ/lin*ANRIL* and the proliferative index in islet cells may be a consequence of increased circ*ANRIL* expression shifting the balance between the two RNA species. Since pancreatic beta cell number is an important determinant of insulin secretory capacity^63–65^, and proliferation is the primary mechanism generating new beta cells in the adult pancreas^66^, these observations suggest the possibility that the ratio of circ*ANRIL* to lin*ANRIL* may be one determinant of diabetes susceptibility.

Single nucleotide polymorphisms (SNPs) at the *ANRIL* locus associated with diabetes susceptibility have also been previously associated with β-cell proliferation^12^. Thus, we wondered whether islet circ*ANRIL* abundance was associated with genotypic diversity across individuals and if the relationship between circ/lin*ANRIL* expression and proliferation was mediated by a genetic regulatory pathway. To do so, we genotyped 4 T2D-associated SNPs at the *ANRIL* locus (rs564398, rs2383208, rs10757283, rs10811661) across our expanded cohort of islet preparations. Excitingly, we observed a significant association between circ/lin*ANRIL* abundance and genotype at rs564398 (Fig. 4D), which is located within *ANRIL* exon2. Specifically, the T2D risk (T) allele at rs546398 was associated with higher circ/lin*ANRIL* abundance. The other 3 T2D associated SNPs, located downstream of the gene region, were not associated with circ/lin*ANRIL*. We did not see significant correlations between rs564398 genotype and the expression of either lin*ANRIL* or any circ*ANRIL* isoform independently (Fig. S4C). The previously observed association between the homozygous protective CC genotype and higher beta cell proliferation^12^ is also consistent with this genotype being associated with lower circ/lin *ANRIL*.

### Back-splicing is ubiquitous in pancreatic islets

**Figure 5:**
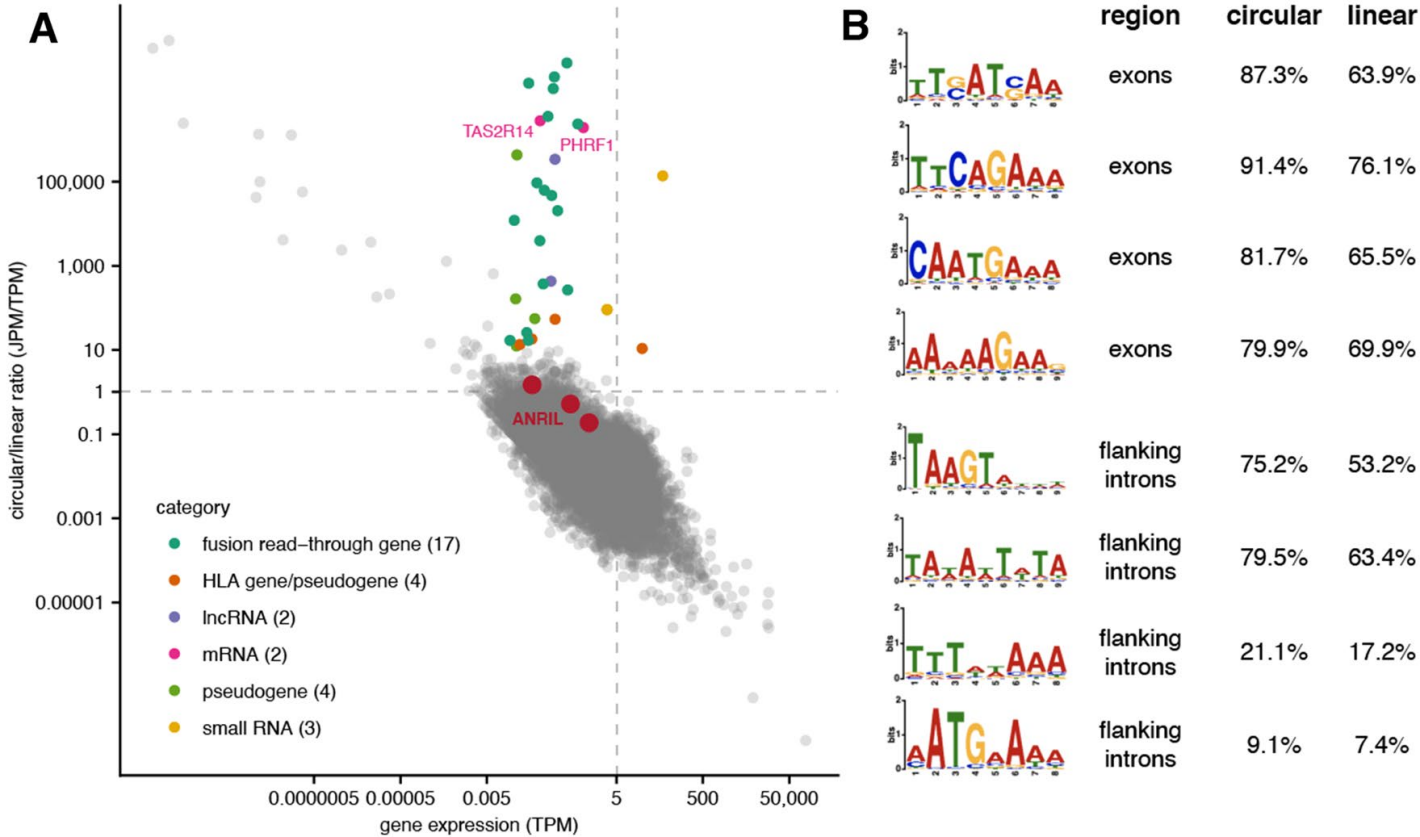
Global expression of circular RNAs in pancreatic islets. (**A**) Correlation between linear gene expression (TPM, *x-axis*) and circular/linear ratios (JPM/TPM, *y-axis*) for all genes with circRNAs in pancreatic islet cells. Genes that diverge from the global correlation patterns are highlighted, with colors differentiating by gene type or functional category. Dotted lines indicate equal circular/linear abundance (*horizontal*) and a threshold for expressed genes (*vertical*). (**B**) Sequence motifs enriched in circRNA-involved exons or flanking introns, with percent occurrence in circular or non-circular (linear) regions indicated on the right.

To evaluate our observations about circ*ANRIL* expression within the broader context of back-splicing regulation, we looked at the global distribution of circRNA expression in human islets (Table S8). A previous study, Haque et al. 2020, found that circRNAs are abundantly expressed in human pancreatic islets, including circ*CIRBP*, circ*ZKSCAN*, circ*RPH3AL*, and circ*CAMPSAP1*, which were also associated with diabetes status and specific diabetes relevant islet cell phenotypes^37^. Of these, we found that circ*CIRBP* and circ*ZKSAN* among the top 15% highest expressed circRNAs on average in our data. However, we note that only one of the 5 pancreatic islet samples on which we performed high-throughput sequencing was from a diabetic individual, limiting our power to identify circRNAs associated with diabetes status. Furthermore, we only looked at RNA expression in resting islets, unlike the Haque et al. study. Consistent with previous studies^67^, we found a strong negative correlation between the circular/linear ratio and linear gene expression across most genes (Spearman R = − 0.6; Fig. 5A; Table S9). However, there was a notable cluster of genes that deviate from the global trend, with moderately low linear expression but relatively high circular/linear ratios. While most of the transcripts derived from these loci are either fusion read-through transcripts, pseudogenes, HLA cluster genes, or non-coding RNAs, there are two low abundance mRNAs represented in this cluster: *TAS2R14* and *PHRF1*. Interestingly, *TAS2R14* is a bitter taste receptor family member that has been shown to stimulate GLP-1 secretion, leading to reduced β-cell apoptosis and increase in β-cell proliferation^68–71^. Finally, testing for enriched sequence motifs in exons involved in back-splicing or intronic regions flanking back-spliced exons (Methods) did not reveal any motifs that overlap those enriched in *ANRIL* back-spliced exons (Figs. 5B; S5A). This potentially suggests that *ANRIL* circularization is regulated in a locus-specific fashion rather than by global patterns. However, we did observe a number of motifs that are significantly enriched in circularized exons and respective flanking intronic regions, suggesting that there may be cis-elements that are shared between back-splicing events and regulated by common trans-factors.

## Discussion

In recent years, both linear and circular forms of the non-coding RNA *ANRIL* have been implicated in cardiometabolic disease. While studies have probed the involvement of *ANRIL* in cardiovascular disease susceptibility and phenotypes^17,20,21^, less is known about how *ANRIL* plays a role in T2D, despite multiple strongly validated T2D risk polymorphisms within or near this gene locus^13^. Building on our prior observation that T2D risk-SNPs were associated with increased *ANRIL* abundance and decreased beta cell proliferation index, in this study we used high-throughput sequencing to systematically characterize the identity and abundance of circ*ANRIL* isoforms in human pancreatic islet cells. We are able to identify a diversity of moderately abundant circ*ANRIL* isoforms that are consistently expressed in islets, many of which have been previously identified in other cell types and characterize features that may regulate circularization in islets. Our most important findings include the characterization of features that may regulate *ANRIL* circularization in islets and association of circ/lin *ANRIL* ratios with cellular proliferation and an exonic T2D-associated risk SNP.

Previous studies seeking to identify circ*ANRIL* molecules have mostly used Sanger sequencing after divergent primer PCR^17,18,20,30,40^, which limits the detection of isoforms due to primer selection and placement. Our unbiased high-throughput sequencing approach allowed us to identify multiple distinct isoforms for circ*ANRIL*, with extensive alternative splicing leading to varied internal exon structures. Our results expand the repertoire of isoforms and the exonic or intronic sequences that could be involved in regulation of or by circ*ANRIL*. While other studies in immune or cancer cells have mostly focused on one predominant isoform^17,18,20^, we observe that at least 5 isoforms are relatively abundant in islets. This includes isoforms 7–5, 10–5, and 16–5, which were previously identified and characterized in blood monocytes^17^ and T lymphocytes^20^ using clonal sequencing. Our observations may reflect cell type heterogeneity, where each individual cell type within islet populations may have a specific predominant isoform, or point to extensive intra-cellular circ*ANRIL* diversity that was underappreciated in previous studies.

Across all circ*ANRIL* isoforms in islets, a clear pattern emerged that back-splicing mostly involved a specific set of splice sites: 5’ splice sites from exons 7, 10, and 16 and 3’ splice sites from exons 2, 4, and 5. These observations suggest that back-splicing does not occur with equal probability at every *ANRIL* splice site, but rather, features of these exons or splice sites may promote back-splicing. While we and others^18^ see small differences in intron length, sequence complementarity, and motif enrichment for genomic regions flanking these splice sites, there is no one primary differentiating feature. It appears that the frequency of back-splicing is regulated, since circular isoform expression was correlated across individuals. Notably, the abundance of circ*ANRIL* isoforms were more correlated with each other than with lin*ANRIL* or other mRNAs transcribed from the locus, suggesting independent, non-transcriptional regulation of these distinct *ANRIL* molecules. Previous studies have also seen that overall circ*ANRIL* abundance may be more correlated with *CDKN2A* and *CDKN2B* isoforms and perhaps even regulate the abundance of these mRNAs^30^. Together, these results highlight the need for a greater understanding of circ*ANRIL* regulation across different cell types.

While there are many proposed potential functions for circRNAs, one enduring idea is that circRNAs act as sponges that sequester miRNAs away from their intended targets^72,73^. For this to occur, the circRNA must have target sites for the specific miRNA, with an enrichment of target sites relative to the frequency expected by chance. A previous unbiased bioinformatic analysis trying to identify miRNA sites in circ*ANRIL* only identified 3 miRNA target sites in circ*ANRIL*^20^. However, this study focused solely on isoform 7–5, which only has 3 exons and is expressed almost at the same level as other isoforms containing many more exons in islet cells. Using these longer isoforms, we are able to identify many more miRNA target sites across exons involved in circ*ANRIL* and/or lin*ANRIL*. Notably, we find that exons involved in circ*ANRIL* have a fourfold enrichment of target sites for miR-130-5p, which has been previously implicated in pancreatic cancers. More work is needed to evaluate the direct binding of the bioinformatically enriched miRNAs we identify and the role of circ*ANRIL*-miRNA interactions in islet cells. While previous studies have experimentally characterized miRNA binding to both linear and circular *ANRIL* molecules in various cell types and cellular contexts^55–58^, often the effect is detectable only after cellular stimulation or injury. Similarly, other prominent circRNAs thought to act as miRNA sponges, like CDR1as, have stronger effects after cellular stimulation in islet cells^74^. Indeed, a previous study of circRNAs in pancreatic islets also found that several diabetes-associated circRNAs showed higher expression and association with cellular phenotypes after cellular stimulation^37^. Our analyses were performed on pancreatic islets under basal conditions.

Intriguingly, we find that the ratio of circular to linear *ANRIL* abundance is associated with beta cell proliferation index and with genotype variation at a T2D-associated SNP. The balance between linear to circular *ANRIL* species has been previously implicated in regulating the balance between proliferative and apoptotic phenotypes in atherosclerosis models^17,20^. That study proposed a model for cardiovascular disease, in which increased atherosclerosis risk is associated with increased linear *ANRIL*; linear *ANRIL* epigenetically regulates factors that promote cell adhesion and proliferation by acting as a molecular scaffold for the polycomb repressive complex (PRC1/2). In contrast, they posit that protection against atherosclerosis is associated with increased circ*ANRIL*, where circ*ANRIL* protects against over-proliferation by binding and impairing PES1 function to impede ribosomal RNA maturation^20^. Similar effects were observed in human brain microvascular endothelial cells, in which circ*ANRIL* overexpression inhibited proliferation and apoptotic phenotypes^57^ These effects were the most pronounced upon cellular stress. Our observations suggest that the balance between circ*ANRIL* and lin*ANRIL* may similarly play a role in regulating proliferation of pancreatic beta cells.

Our study has several limitations. Our RNA sequencing and gene expression analyses were performed on whole pancreatic islets, which contain endocrine cells including alpha, beta, delta, and other cells, as well as non-endocrine cells such as endothelial cells. The cell-type distribution of the *ANRIL* isoforms studied has not been assessed. However, our major results were validated in the endoC-βH1 cell line, an established fetal human beta cell line^39^. The use of this cell line provides a complementary cell population to primary pancreatic islets that is likely composed of a more homogeneous β cell population. The insulin secretion analyses included here were based on glucose stimulated insulin secretion testing performed on-site at each human islet isolation center^12^; inter-site variation, as well as the high individual variation already noted in human islet preparations^75^ may have obscured findings. Furthermore, the majority of samples analyzed here are from non-diabetic individuals. The expression of both linear and circular *ANRIL* isoforms may be quite dynamic in individuals with T2D or under diabetes-related stresses. It is even possible that different *ANRIL* isoforms are expressed under these conditions. Finally, despite a combined high-throughput and targeted approach to identify and quantify circ*ANRIL* isoforms in islet cells, we were limited by the relatively low abundance of *ANRIL* RNAs. The need for RNase R to enrich for circRNA and a paucity of BSJ reads across samples hindered our ability to quantify *ANRIL* isoforms at a high resolution. Techniques to overexpress this locus—either through in-vivo stimulation or experimental perturbation—may prove useful in alleviating this issue.

In sum, our work reveals exciting new biology of the *ANRIL* lncRNA, including detailed exploration of its circular isoforms in primary human pancreatic islet tissue. Future studies are needed to dissect the regulation of *ANRIL* circularization, as well as the molecular mechanisms by which linear and circular *ANRIL* molecules influence beta cell proliferation under basal, stimulated, and stress conditions. Whether *ANRIL* isoforms impact other important parameters of islet function such as glucose sensing, insulin secretion and cell survival under stress remains unknown. Given the marked worldwide increase in T2D and associated social costs, and the robust relationship between *ANRIL* locus SNPs and T2D risk, further studies are warranted.

## Materials and methods

### Human pancreatic islet preparations

Human pancreatic islets were obtained from the Integrated Islet Distribution Program (IIDP) at the City of Hope, supported by the National Institute of Diabetes and Digestive and Kidney Diseases (NIDDK), National Institutes of Health, or from a collaborative group headed at Vanderbilt University^76^. Human islet studies were determined by the University of Massachusetts Institutional Review Board to not qualify for institutional review board review or exemption because they do not involve the use of human subjects.

De-identified islet samples from 112 subjects without diabetes and 10 subjects with T2D were live shipped in Prodo islet transport media. Human islets were cultured overnight in islet culture medium for recovery from isolation and shipment, then were handpicked and flash frozen at − 80 °C as previously described^12^. DNA and RNA from the frozen islets were extracted using the Norgen RNA/DNA/Protein Purification Kit (Norgen Biotek Corp. Thorold, Ontario, Canada).

### RNA sequencing from islet preparations

RNA from 5 frozen islet donor preps acquired via the IIDP was isolated using TRIzol (15596018, Thermo Fisher Scientific). All RNA integrity numbers (RIN), as measured on a 4150 Tapestation System using a High Sensitivity RNA ScreenTape Assay kit (5067-5579/5580, Agilent Technologies), were greater than 8. Ribosomal RNA depletion was performed with the Illumina TruSeq Ribo-Zero Gold rRNA Removal kit (MRZH116, Illumina). Following the method in^77^, rRNA depleted islet RNA was digested using RNase R (RNR07250, Lucigen) or mock digestion (protocol without RNase R) followed by clean-up with the Qiagen RNeasy Mini kit (Q74106, Qiagen). RNA-seq libraries were prepared with the TruSeq Stranded Illumina Total RNA Preparation Kit (20020599, Illumina). Paired-end 2 × 150nt sequencing of libraries was performed in-house on the Illumina NextSeq 550 sequencer using a NextSeq 500/550 300 cycle High Output Kit v2.5 (20024908, Illumina).

### Analysis of RNA-seq data and gene expression

Quality control for the RNA-seq data was performed using FastQC, files were filtered for low quality reads, and adapters were trimmed using trimmomatic/0.3.2^78^. Data were mapped to the hg38 reference genome^16^ using STAR/2.7.0e^36^, guided by gene annotations from ENSEMBL hg38.v95. Data from Haque et al.^37^ were downloaded from the NCBI BioProject Database (accession number PRJNA607015) and mapped as described above. Linear RNA gene expression levels were measured using TPMs generated by kallisto/0.4.0^79^.

### Identifying and quantifying circular RNAs from RNA-seq data

Back-splice junctions (BSJs) were first identified in the RNA-seq data with the CIRCexplorer2^35^ characterization pipeline. The CIRCexplorer2 Parse module was run with default parameters using chimeric alignment output files created by using the –chimOutType flag when running STAR to create back-splice junction files. Sashimi plots in Fig. 1A were generated using the following data: (1) normalized base-specific read counts per strand, generated with igvtools count, (2) linear splice junction reads reported in the sj.out.tab file from the STAR aligner, and (3) back-spliced junctions reported by CIRCexplorer2. To quantify circRNA abundance, we use the RNase R effect correction pipeline in the CIRIquant package^38^. This package provides the ability to perform a direct comparison between linear and circular RNA species and accounts for both RNase R and untreated samples to calculate an adjusted BSJ count per locus. High-confidence BSJs were defined as those present in more than one individual and/or supported by more than one junction read.

The *ANRIL* meta-isoform was created by merging all overlapping isoform annotation coordinates for linear CDKN2B-AS1 isoforms in ENSEMBL v95. Adjusted BSJ reads from CIRIquant were counted and attributed to annotated *ANRIL* exons based on splice site usage. HTseq/0.1.0^80^ was used to count exonic coverage across RNase R and untreated samples.

To quantify the total expression of circRNAs within each sample, we calculated a junctions per million mapped reads (JPM) metric, based on the principle that each BSJ read could only be derived from a single circRNA molecule: ${\varvec{JPM}} = \user2{ }\frac{{\# \user2{ BSJ\, reads}}}{{\# \user2{ mapped\, reads } \times10^{6} }}$.

### Sequencing validation of ANRIL circular RNAs

The human immortalized β cell line, endoC-βH1, was cultured as previously described and passaged every 7 days^39^. Cells were grown in culture vessels coated with 2 µg/ml Fibronectin (F1141, Sigma-Aldrich) and 1% extracellular matrix (ECM) (E1270, Sigma-Aldrich) mixed in high glucose DMEM (11,965–092, Life Technology) and cultured in low glucose DMEM containing 5.5 mM glucose (11,885–084, Life Technology), 2% bovine serum albumin (BSA) (BAH66, Equitech), 10 mM nicotinamide (481,907, VWR), 100 U/ml penicillin, 100 µg/ml streptomycin (P/S) (P4333, Sigma-Aldrich), 50 μM β-2-mercaptoethanol (M3148, Sigma-Aldrich), 5.5 μg/ml transferrin (T8158, Sigma-Aldrich) and 6.7 ng/ml sodium selenite (S1382, Sigma-Aldrich).

Total RNA was isolated from endoC cells using the Norgen RNA/DNA/Protein Purification Kit (Norgen Biotek Corp. Thorold, Ontario, Canada) and subjected to reverse transcription using the SuperScript IV VILO MasterMix kit (Thermo Fisher Scientific) according to the manufacturer’s protocols. Outward facing primers were used against *ANRIL* exons 2, 3, 4, 6, 7, 8, 16 and 19 with forward primer against the 3′ end of each exon and reverse primer against 5′ end of the same exon (Table S3). PCR reactions were performed following the cycling conditions: 95 °C for 5 min, 35 cycles (95 °C for 30 s, 59 °C for 30 s, and 68 °C for 1 min), and 68 °C for 5 min. PCR products were cloned using the TOPO TA Cloning Kit (Life Technologies). The positive clones were selected by digesting the purified clones with XhoI and HindIII restriction enzymes and sequenced using M13 Forward (− 21) and M13 Reverse primers performed in two directions. The sequencing results from the clones were aligned to each exon sequence of *ANRIL* to identify the circular *ANRIL* junctions and isoforms.

### qRT-PCR quantification of linear and circular RNA

Total RNA was reverse transcribed using a SuperScript IV VILO MasterMix kit (Thermo Fisher Scientific). For relative expression of circ*ANRIL* junctions in EndoC cells and human islet samples by qPCR, Taqman primer and probe sets for the detection of *ANRIL* junctions 7–5, 10–5, 16–5, and 16–4 were designed. Together with two commercial TaqMan *ANRIL* expression assays *ANRIL* (Exon5-6), Hs04259476_m1; and *ANRIL* (Exon1-2), Hs01390879_m1, as well as those for *CDKN2B* p15INK4b, Hs00793225_m1; *CDKN2A* p16INK4a, Hs02902543_mH; *CDKN2A* ARF, Hs99999189_m1, the expression levels of target genes were quantitatively assessed in duplicate by normalizing to the level of endogenous reference (*ACTB*, Hs01060665_g1; and *GAPDH*, Hs02758991_g1) Transcript expression levels were presented as log2-transformed expression (ΔCt).

#### RNase R treatment for qPCR

Equal amounts of RNA (2 µg) were incubated with or without 20 U of Rnase R (RNR07250, Epicentre Biotechnologies) and 40 U of RiboLock RNase inhibitor (EO0381,Thermo Fisher Scientific) in a 20µL reaction volume for 30 min at 37 °C. The resulting RNA was purified as described above and quantified. Equal volumes of RNA were then subjected to reverse transcription using the SuperScript IV VILO MasterMix kit (Thermo Fisher Scientific, #11,756,050) with a mixture of random hexamer and oligo dT primers. qPCR was performed to quantify the transcripts using Taqman primers/probes for *ANRIL* exon1-2, *ANRIL* exon5-6, *ANRIL* back-spliced junctions (7–5, 10–5, 16–4 and 16–5). *Actin* and *GAPDH* were used as controls. The mRNA depletion was examined by normalizing the relative expression of transcripts with RNase R treatment to the untreated control.

#### Isolation of cytoplasmic and nuclear RNA

EndoC cells were collected for subcellular fractionation and total RNA isolation. Cytoplasmic and nuclear RNA were isolated with the Cytoplasmic & Nuclear RNA Purification Kit (Norgen, Belmont, CA, USA) following the manufacturer’s manual. Briefly, EndoC cells were harvested and incubated with a lysis buffer for 5 min on ice. Then, the cells were centrifuged at maximum speed for 10 min at 4 °C, the supernatant was kept for assessing the cytoplasmic RNA, and the pellet was used for nuclear RNA extraction. Next the RNA was reversely transcribed to cDNA according to the instructions of SuperScript IV VILO MasterMix kit (Thermo Fisher Scientific). The expression levels of *ANRIL* (Exon5-6), Hs04259476_m1; *ANRIL* (Exon1-2), Hs01390879_m1, circular *ANRIL* (7–5, 10–5, 16–4 and 16–5) in the whole cells, nuclei and cytoplasm were examined by qRT-PCR. Glyceraldehyde 3-phosphate dehydrogenase (*GAPDH*) and Nuclear Enriched Abundant Transcript 1 (*NEAT1*) were detected as fractionation indicators. The primers for *GAPDH* RNA were 5'-CTCCTCCTGTTCGACAGTCA-3' (sense) and 5'-GTTGACTCCGACCTTCACCT-3' (antisense). The primers for *NEAT1* RNA were 5'-GTGGCTGTTGGAGTCGGTAT-3' (sense) and 5'-TAACAAACCACGGTCCATGA-3' (antisense).

### Sequence characteristics for ANRIL exons and introns

#### Splice site scores

The strength of splice sites for *ANRIL* exons were calculated using a maximum entropy model as implemented in maxEntScan^81^ using 9nt around the 5’ splice site (− 3: + 6) and 23nt around the 3’ splice site (− 20: + 3).

#### Repeat regions

Repeat annotations for all repeat classes were downloaded from RepeatMasker (www.repeatmasker.org^82^) and overlapped with *ANRIL* meta-exon and intervening intronic coordinates to look for enrichments within regions involved in or proximal to back-splicing events.

#### Sequence complementarity

*ANRIL* exon and intron sequences were extracted based on the *ANRIL* meta-isoform coordinates and inverted complementary sequences were created for each region. All pairwise combinations of exon-exon or intron-intron (for upstream or downstream flanking introns, independently) were evaluated for sequence complementarity using blastn (parameters -word_size 7 -gapopen 5 -gapextend 2 -penalty -3 -reward 2) similar to described previously^46^. Pairs of regions seen to interact in BSJs were compared to an equal number of randomly selected non-occurring pairings.

### Motif analyses

*ANRIL* exon and intron sequences were extracted based on the *ANRIL* meta-isoform coordinates. For exons, full exonic sequences were used, but introns were split into two equal regions proximal or distal to the 3’ or 5’ splice site involved in the back-splicing reaction and proximal regions were only compared to similarly defined proximal regions within the splice site type. For example, intronic regions proximal to BSJ-involved 3’ splice sites were compared to intronic regions proximal to 3’ splice sites not involved or minimally involved in BSJs. Motif analyses were conducted using STREME^83^ to identify enriched sequence motifs in BSJ-involved exons or proximal intron regions relative to exons or intronic regions with no or low evidence for back-splicing.

To perform similar motif analyses across the full set of circRNAs expressed in islets, we first considered genes with a TPM higher than 5 measured by kallisto/0.4.0 and identified expressed exons as those with junction read counts in the bottom 10% of the distribution of junction counts as reported by STAR/2.7.0e. Expressed exons that matched BSJ coordinates from CIRIquant were probed for enriched sequence motifs using STREME, with expressed exons not matching BSJ coordinates used as a background set. For intronic motif enrichments, we used sequences 100 bp upstream of 5’ splice sites and 100 bp downstream of 3’ splice sites. Enriched motif sequences for all of the above comparisons were compared with the RNA Binding Protein Database (RBPDB; http://rbpdb.ccbr.utoronto.ca/) to identify candidate RNA binding proteins that bind to enriched motifs.

### miRNA analyses

To define full length circ*ANRIL* isoforms, we considered as circular any exon present in at least 10% of clones sequenced using Sanger sequencing. We used miRanda 3.3a^84^ to predict miRNA target sites in *ANRIL* exons, only considering miRNAs with a mean expression in islet cells greater than 5 reads per million based on three datasets downloaded from miRmine (https://guanfiles.dcmb.med.umich.edu/mirmine/index.html): SRX290576, SRX290601, SRX290617). Finally, we normalized the number of miRNA sites in each exon category (linear or circular) by the total length of the exons.

### Cellular phenotype assays

#### Proliferation assays

Human islets from a subset of donors (n = 45) were cultured overnight in islet culture medium, trypsinized to single cells using and plated on uncoated glass coverslips (Fisherbrand) as previously described in Kong et al. 2018^12^. Dispersed cells were cultured in islet culture medium containing either 5 or 15 mmol/L glucose and 20 μg/mL bromodeoxyuridine (Sigma) for 96 h. After culture, the islet cells were fixed for 10 min in 4% paraformaldehyde (Sigma). Fixed cells were unmasked in 1 N HCl for 20 min at 37 °C, blocked for 2 h in goat serum–based block with 0.1% Tween 20. Immunofluorescence staining was performed for insulin (ab7842, Abcam, or A056401-2; Dako), and BrdU (ab6326; Abcam) antibodies, and DAPI (Sigma) as previously described^12^. The percent of insulin-staining cells that were also BrdU labeled, was quantified on blinded images to calculate β–cell proliferation. Representative images of data are shown in Fig. S9 of Kong et al. 2018^12^. Data were expressed as the proliferation index, calculated using the following ratio.

$$proliferation\, index = \frac{{\% \,BrdU\left( + \right) \& \,insulin\left( + \right) \beta \,cells \,in \,15\frac{{{\text{mmol}}}}{{\text{L}}}glucose}}{{\% \,BrdU\left( + \right) \& insulin\left( + \right) \beta \,cells \,in \,5\frac{{{\text{mmol}}}}{{\text{L}}}glucose}}$$

#### Insulin secretion assays

A subset of islet samples (*n* = 83) were tested for glucose stimulated insulin secretion at the time of islet isolation by each individual isolation center; these data were downloaded from IIDP website.

#### Genotyping

Genotyping for four CDKN2A/B SNPs (rs564398 [C/T], rs10811661 [C/T], rs2383208 [G/A], and rs10757283 [C/T]) were performed in the DNAs of human islets using commercial (C_2618017_10, C_31288917_10, C_15789011_10, and C_31288916_10) TaqMan SNP genotyping assays (Thermo Fisher Scientific, Waltham, MA) as previously described^12^.

### Gene ontology analyses

Gene Ontology analyses were conducted by associating each BSJ with an individual Ensembl gene and identifying the following subsets: top 10% and bottom 10% of expressed circRNAs based on JPM values and top 10% and bottom 10% of circular/linear ratios based on JPM/TPM ratios. Background sets were all expressed genes and all expressed genes with circRNAs, respectively. Gene ontology enrichment analyses were performed in an iterative fashion with a custom script to avoid significant gene ontology terms with overlapping gene sets, as described before^85^. *P*-values were computed using a Fisher-exact test and then corrected using a Benjamini–Hochberg multiple test correction.

## Supplementary Information

Supplementary Information 1.

Supplementary Information 2.

Supplementary Information 3.
Table S2. ANRIL BSJs identified from RNA-seq data. Outputs from CIRCexplorer2 (sheet 1) and CIRIquant (sheet 2) for back-spliced junctions in RNaseR treated and untreated samples.

Supplementary Information 4.
Table S4. Sanger sequencing data. Divergent primers used for Sanger sequencing (sheet 1) and sequencing results for each primer set (sheets 2 - 9), listing genomic coordinates for all non-contiguous regions found in individual clones.

Supplementary Information 5.
Table S4. Sanger sequencing data. Divergent primers used for Sanger sequencing (sheet 1) and sequencing results for each primer set (sheets 2 - 9), listing genomic coordinates for all non-contiguous regions found in individual clones.

Supplementary Information 6.

Supplementary Information 7.
Table S6. Target sites identified in ANRIL exons for miRNAs expressed in human islet cells.

Supplementary Information 8.
Table S7. Proliferation measurements for human islet samples. Measurements used to calculate the proliferation index and genotype correlations, including the % of BrdU+ insulin-secreting cells grown with 15 mmol/L glucose and % of BrdU+ insulin-secreting cells grown with 5 mmol/L glucose.

Supplementary Information 9.
Table S8. Global BSJs identified from RNA-seq data. Outputs from CIRCexplorer2 (sheet 1) and CIRIquant (sheet 2) for back-spliced junctions in RNaseR treated and untreated samples.

Supplementary Information 10.
Table S9. Quantification of gene-specific circRNA abundance. CircRNA abundance (in junctions per million) and linear RNA abundance (in transcript per million) for all genes with circRNAs expressed in human islet cells.

## Data Availability

All software used in this study are from open-source collaborative initiatives or public repositories. High-throughput sequencing data have been deposited with the NCBI Gene Expression Omnibus under accession number GSE192541 (https://www.ncbi.nlm.nih.gov/geo/query/acc.cgi?acc=GSE192541).
