# Supplementary material for: High-throughput analysis of ANRIL circRNA isoforms in human pancreatic islets
Source: Sci Rep. 2022 May 11;12:7745. doi: 10.1038/s41598-022-11668-w (PMC9095874; doi:10.1038/s41598-022-11668-w)
Supplement: Supplementary file 1 — Supplementary Information 1. [file 41598_2022_11668_MOESM1_ESM.docx]

**Supplementary Figures**

**
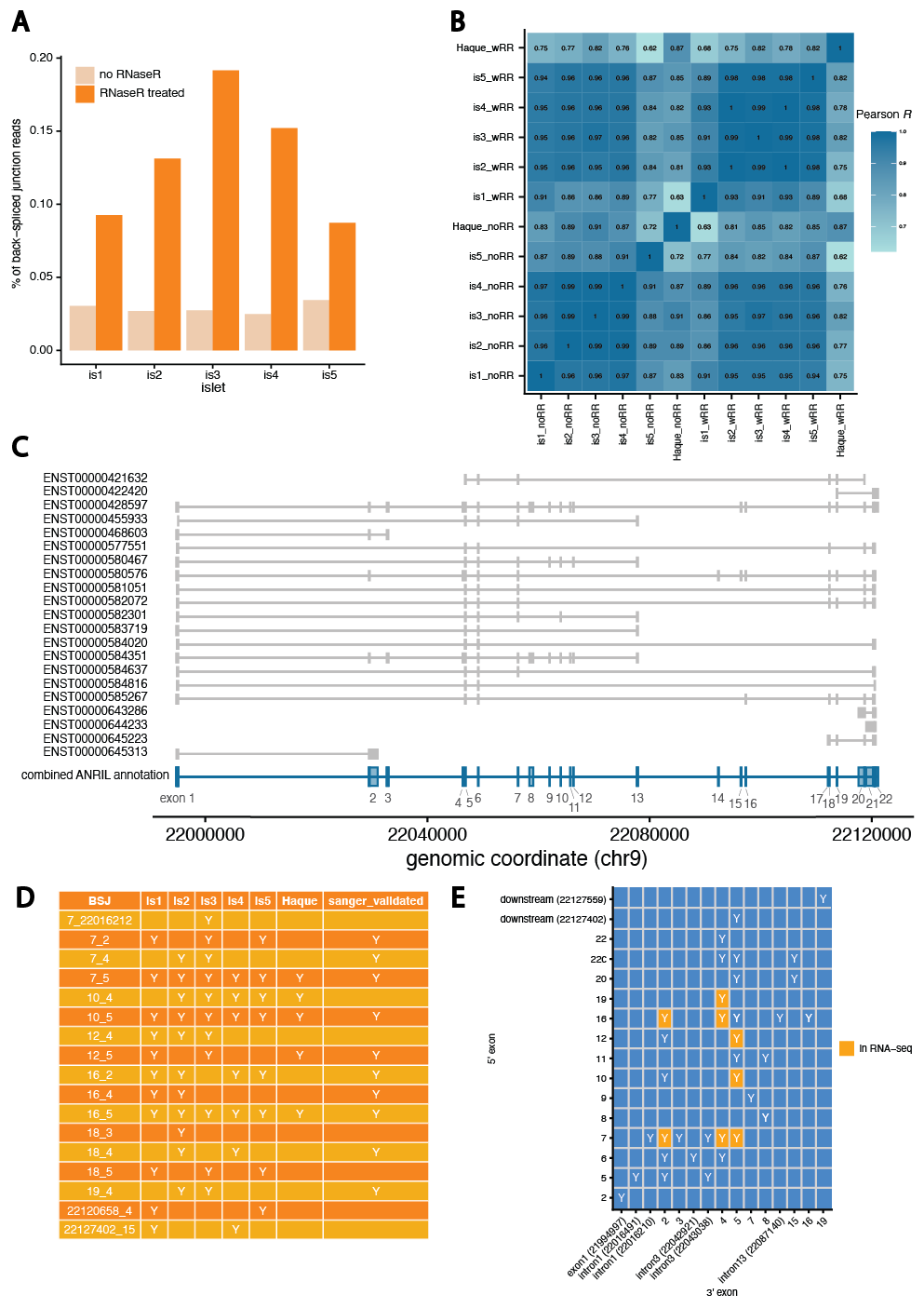
**

**Supplementary Figure 1. Identifying circular *ANRIL* isoforms. (A)** Percentage of reads that represent backspliced junctions across control (*beige*) and RNaseR treated (*orange*) samples from each islet preparation. **(B)** Pairwise Pearson correlations of gene expression levels across samples from 5 islet preparations and published data from Haque *et al*. **(C)** Annotated *ANRIL* isoforms that were combined to create a meta-isoform (*blue*). **(D)** Backspliced junction positions identified from RNaseR RNA-seq samples across each islet preparation and Haque *et al.* data, after filtering for read count and minimum number of samples. **(E)** Backspliced junction positions identified in Sanger sequencing data. Junctions concordant with RNA-seq data are in orange.


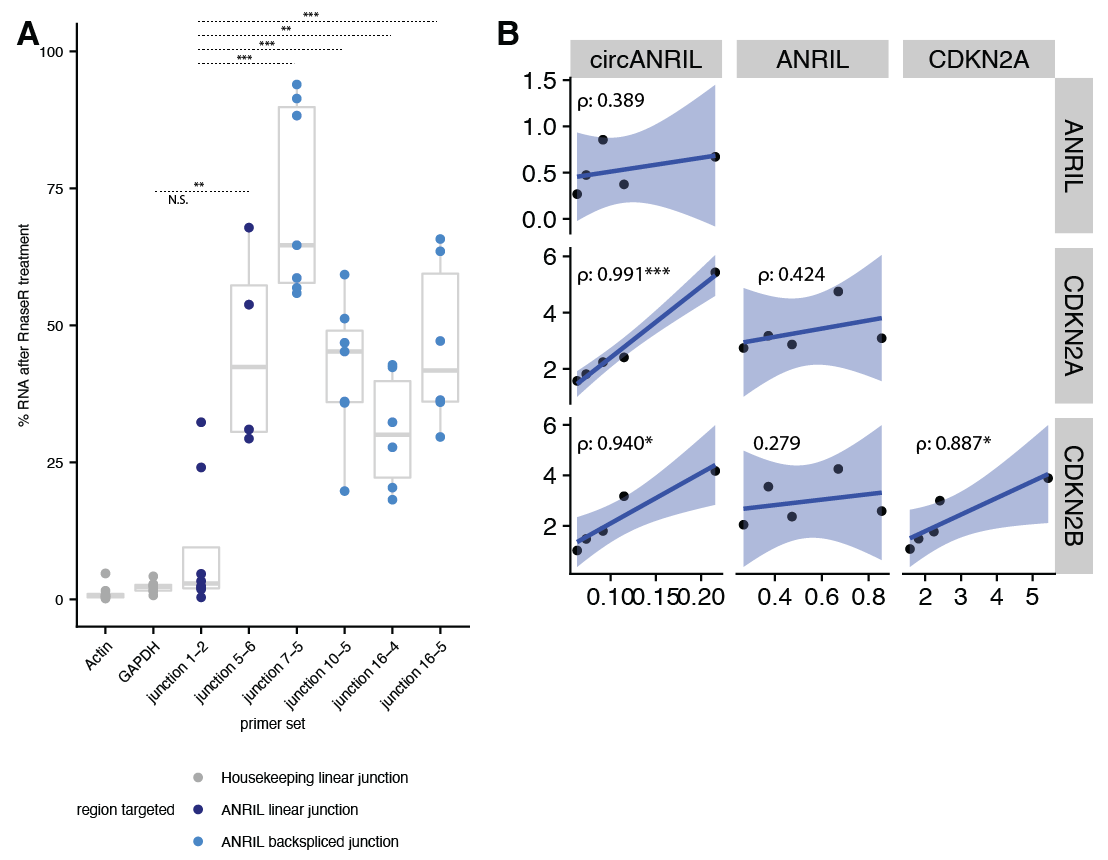


**Supplementary Figure 2. Quantification of *ANRIL* isoforms.** (A) RT-PCR quantification of linear and circular *ANRIL* isoforms in endoC-βH1 cells (** p-value < 0.01, *** p-value < 0.001). (B) Pairwise Spearman correlations between circular *ANRIL* (JPM), linear *ANRIL*, *CDKN2A*, and *CDKN2B* (all TPM) RNA-seq derived expression levels across 5 islet preparations.


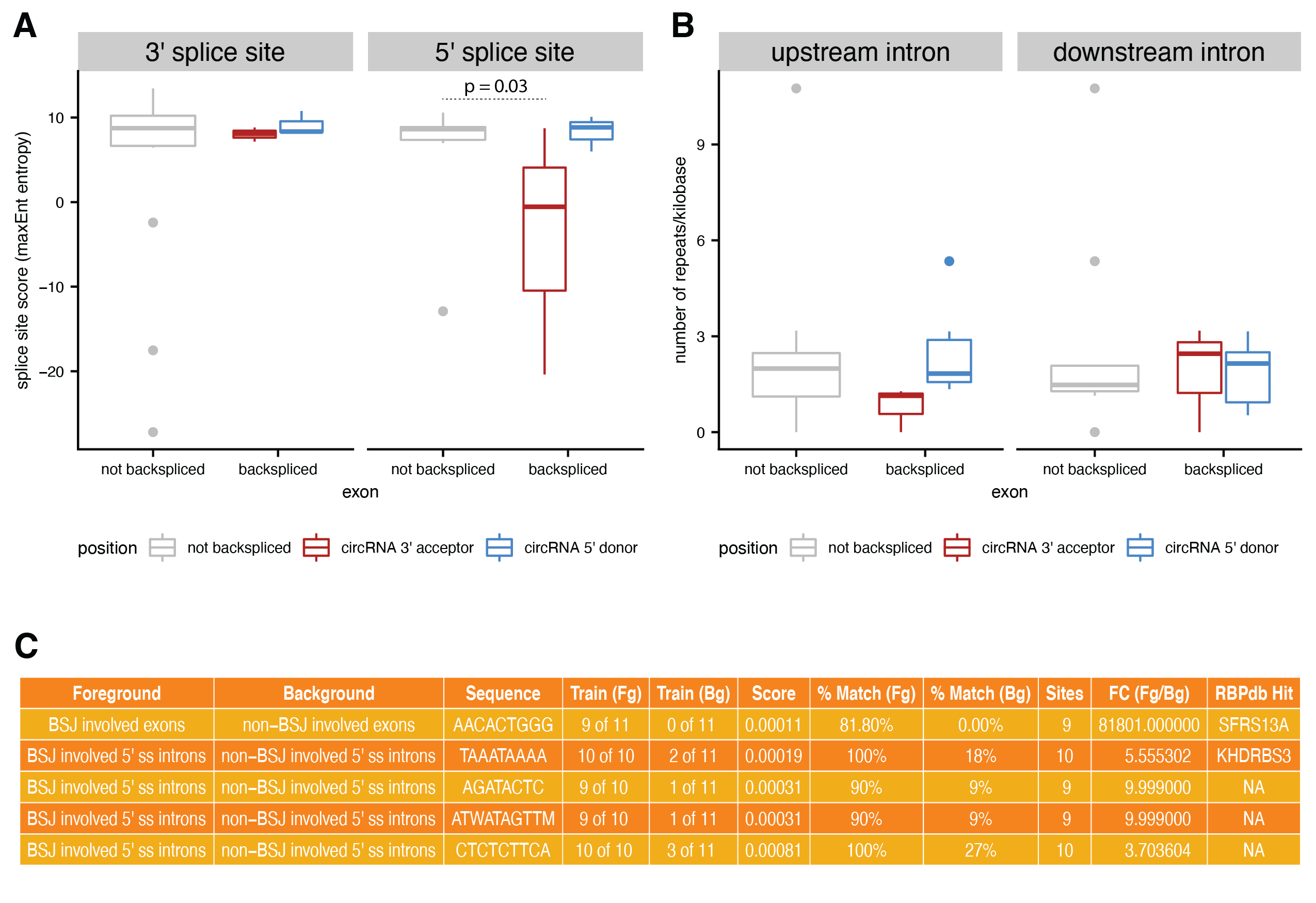


**Figure S3. Regulation of *ANRIL* circRNAs. (A)** 3’ and 5’ splice site maxEnt scores (*y-axis*) for BSJ-involved exons and exons not involved in BSJs. Bootstrapped p-values for all comparisons are not significant, except for the one indicated. **(B)** Distribution of the number of known repeat regions (*y-axis*) in introns located upstream (*left*) and downstream (*right*) of exons involved in BSJs or not involved in BSJs. **(C)** Table of motif enrichments output by STREME showing enriched motifs information and putative RBPs matches from RBPDB. FC indicates fold change between foreground (BSJ-associated) and background (non-BSJ-associated) sequences.


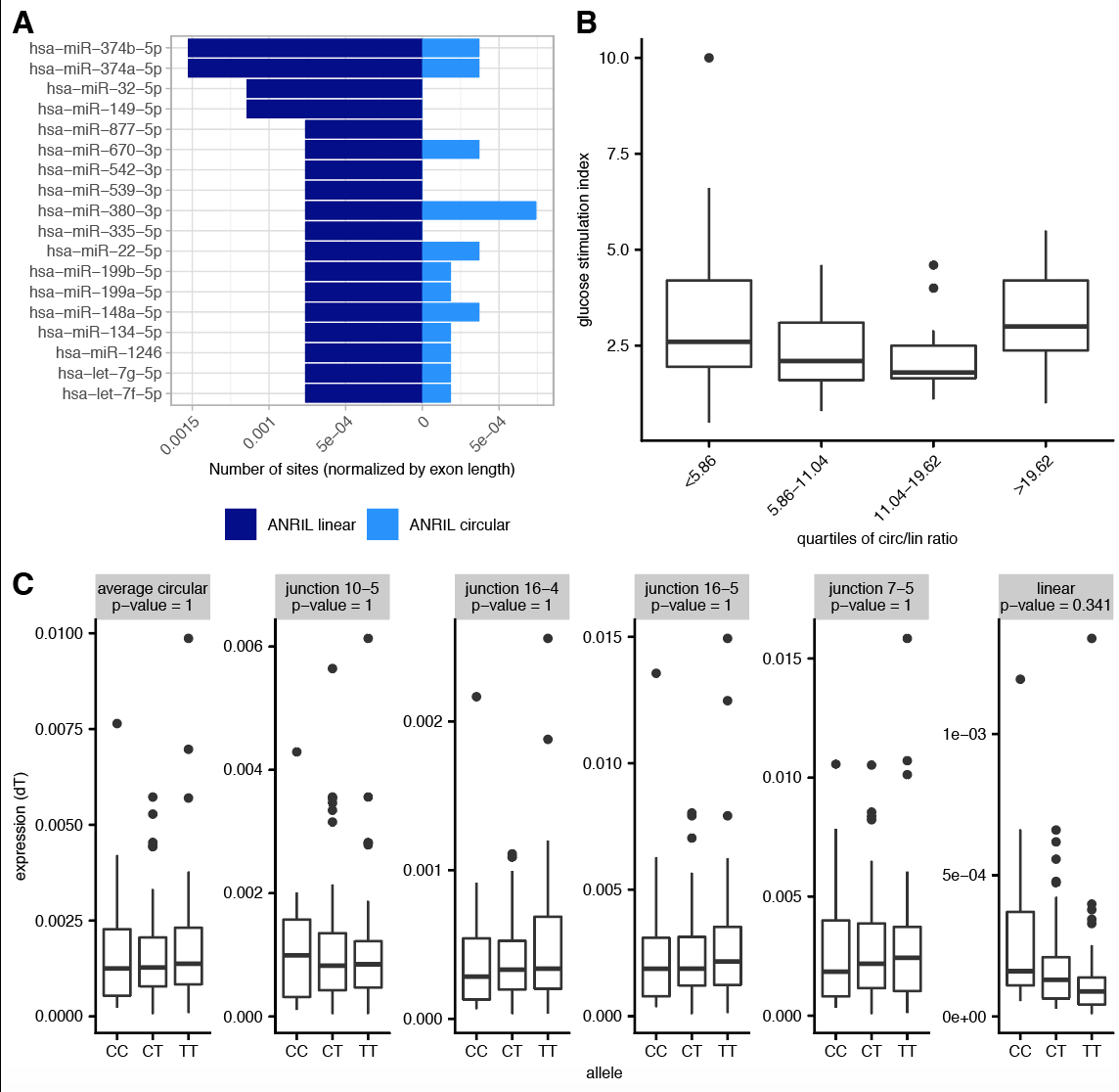


**Figure S4: circ*ANRIL* functional associations. (A)** Number of miRNA target sites (normalized by exon length, *x-axis*) in *ANRIL* circular exons (*light blue*) vs *ANRIL* linear exons (*dark blue*) for miRNAs with more sites in lin*ANRIL* exons. **(B)** Distribution of glucose stimulation indices (*y-axis*) for quartiles of circ*ANRIL*/lin*ANRIL* ratios (*x-axis*) as measured by RT-PCR across 83 islet preparations. **(C)** Association between the genotypes of rs564398 and *ANRIL* isoform expression across 122 islet preparations. P-values are computed from a linear regression and Benjamini-Hochberg corrected.


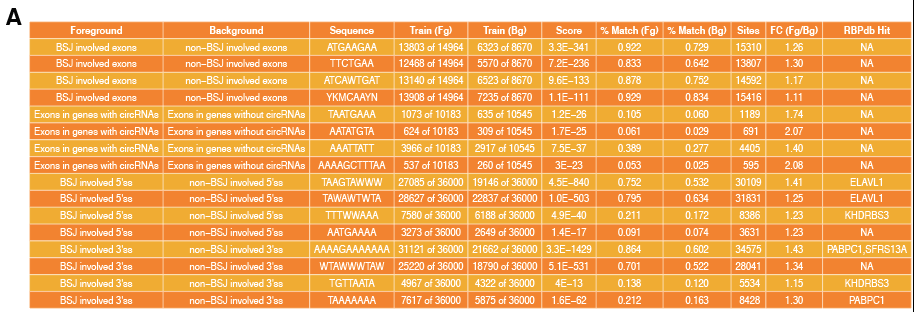


**Figure S5. Global circRNA motif analysis.** Table of motif enrichments output by STREME showing enriched motifs information and putative RBPs matches from RBPDB. FC indicates fold change between foreground (BSJ-associated) and background (non-BSJ-associated) sequences.
